# Supplementary material for: Let-7 enhances murine anti-tumor CD8 T cell responses by promoting memory and antagonizing terminal differentiation
Source: Nat Commun. 2023 Sep 11;14:5585. doi: 10.1038/s41467-023-40959-7 (PMC10495470; doi:10.1038/s41467-023-40959-7)
Supplement: Supplementary file 1 — Supplementary Information [file 41467_2023_40959_MOESM1_ESM.pdf]

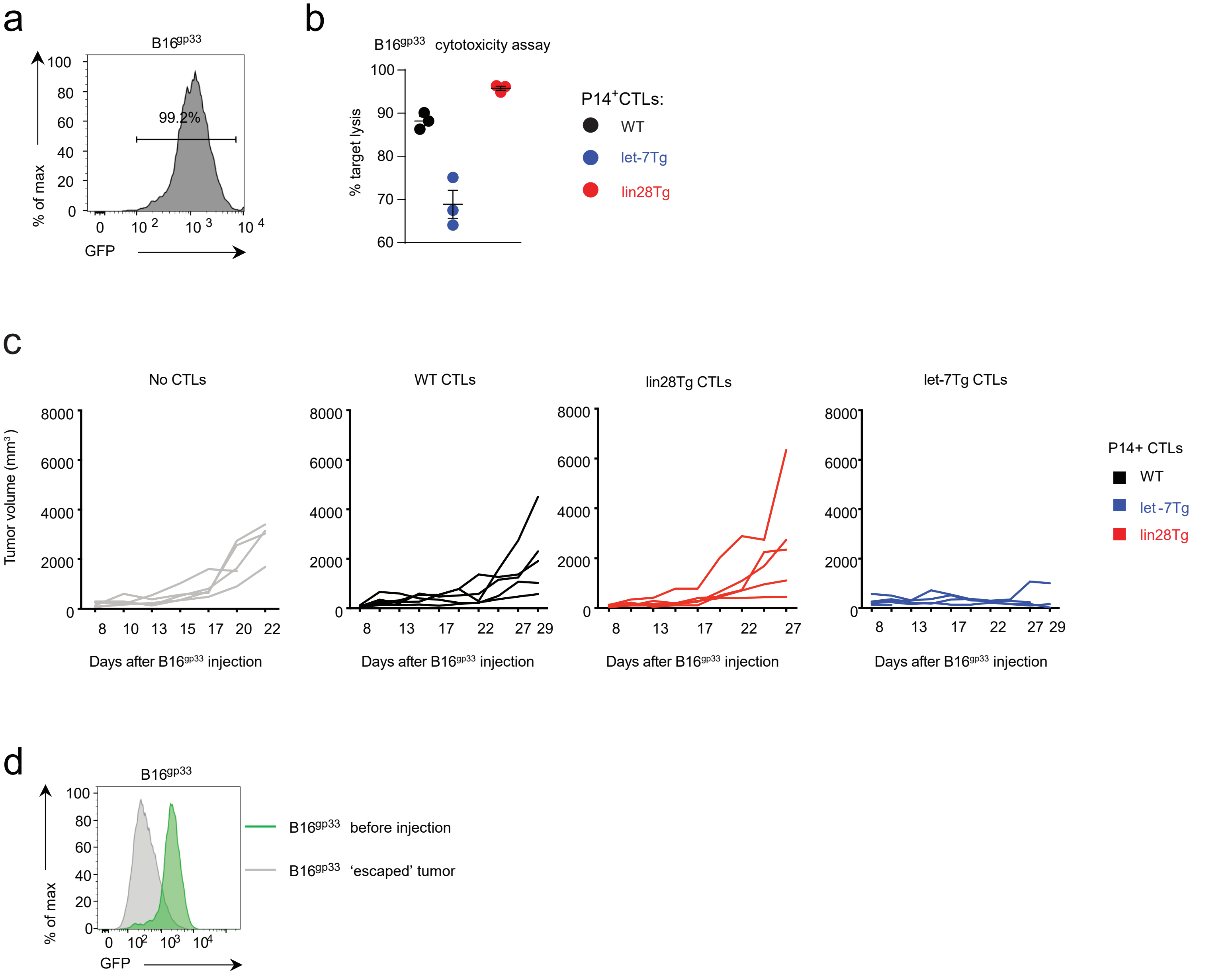

Supplementary Fig. 1

Supplementary Fig. 1.

a, FACS analysis of $B16^{gp33}$ cell line where GFP is reporting expression of gp33 coding mini-gene. **b**, Cytotoxicity assay where $P14^{+}$ CTLs from either WT, lin28Tg or let-7Tg mice (n=3 technical replicates) were cocultured with $B16^{gp33}$ cells. **c**, Individual tumor growth curves in WT mice s.c. injected with $B16^{gp33}$ tumor cells and adoptively transferred with $P14^{+}$ CTLs from either WT, lin28Tg or let-7Tg mice. Grey color represents control group that received no CTLs. **d**, Representative FACS analysis of $B16^{gp33}$ cells before injection and isolated from a tumor from a mouse that received let-7Tg CTLs. Data in **b** represent two independent experiments. Source data for **b**, **c** are provided as a Source Data file.

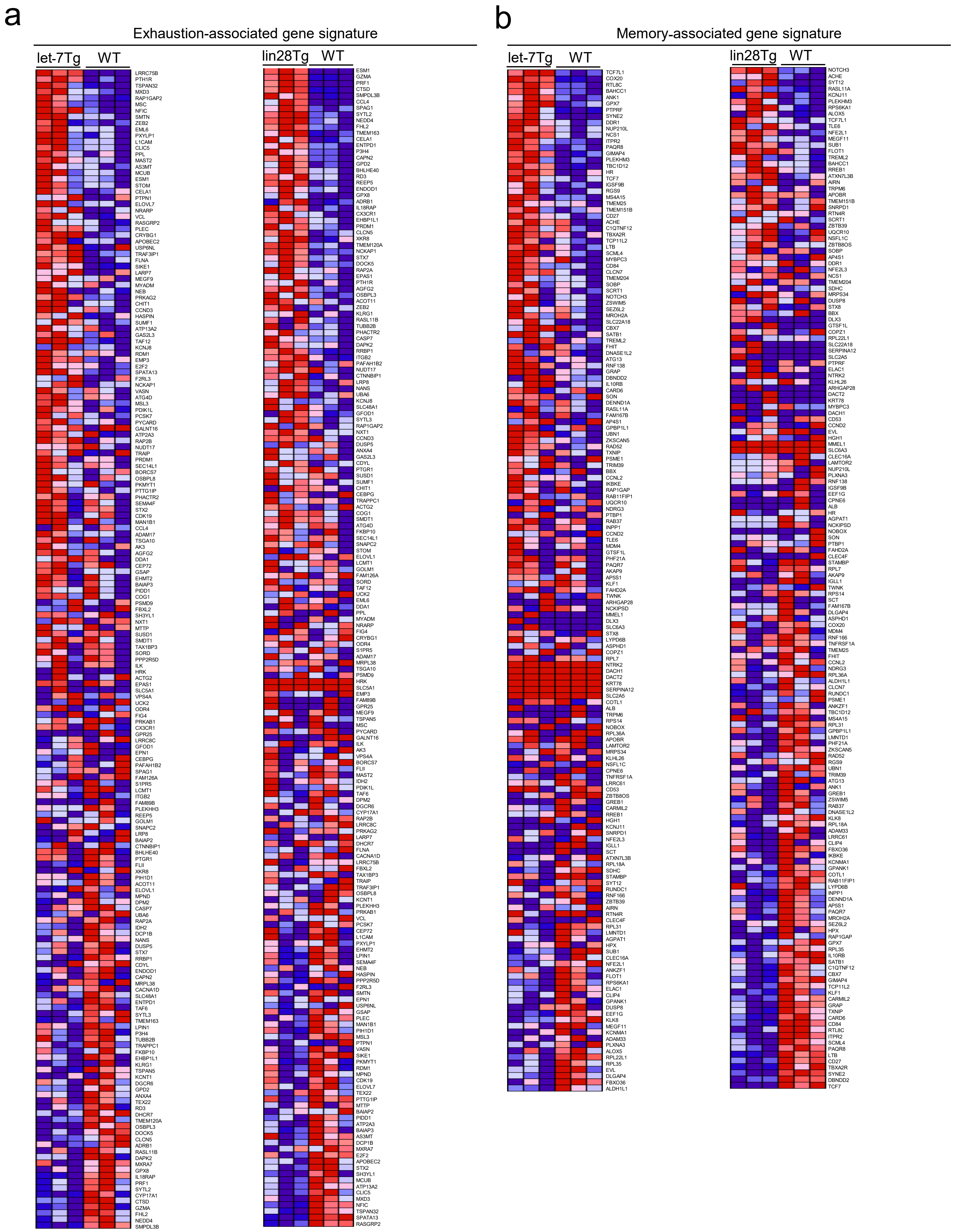

Supplementary Fig. 2

Supplementary Fig. 2.

a-b, Heatmaps of exhaustion-associated genes (**a**) and memory-associated genes (**b**) differentially expressed in P14$^+$ let-7tg and lin28Tg CTLs in comparison to WT CTLs.

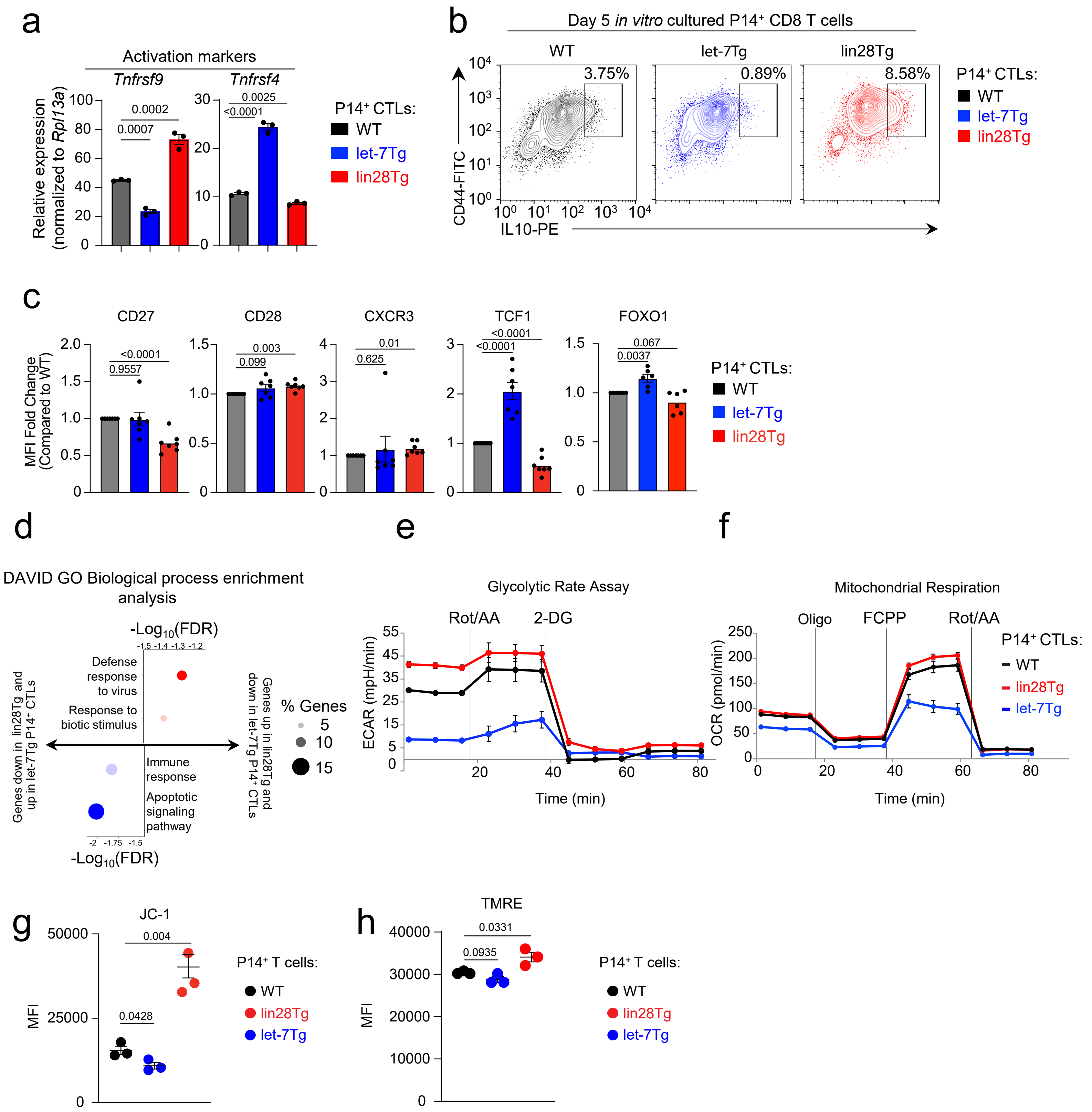

Supplementary Fig. 3

Supplementary Fig. 3.

a, Quantitative RT-PCR analysis showing increased expression of terminal differentiation marker *tnfrsf9* and decreased expression of costimulatory molecule encoded by *tnfrsf4* in lin28Tg CTLs. **b**, Representative FACS analysis of the expression of CD44 and IL-10 in WT, let-7Tg, and lin28Tg CTLs. **c**, Additional FACS of the expression of proteins involved in memory formation in WT, let-7Tg, and Lin28Tg CTLs; quantification of MFIs. **d**, Gene ontology analysis showing selected pathways upregulated and downregulated in $P14^+$ let-7tg and Lin28Tg CTLs. **e**, Extracellular acidification rate of $P14^+$ WT, lin28Tg or let-7Tg CTLs. Rot/AA, rotenone plus antimycin; 2-DG, 2-deoxy-D-glucose, (n=9 technical replicates). **f**, Oxygen consumption rate of $P14^+$ WT, lin28Tg or let-7Tg CTLs. Oligo, oligomycin; FCCP, Carbonyl cyanide-4 (trifluoromethoxy) phenylhydrazone, (n=9 technical replicates). **g**, Quantification of MFIs of mitochondria membrane potential probe JC-1 in WT, let-7Tg, and Lin28Tg CTLs (n=3 technical replicates). **h**, Quantification of MFIs of mitochondria membrane potential probe TMRE in WT, let-7Tg, and Lin28Tg CTLs (n=3 technical replicates). Data in **a**, **e-h** are the mean $\pm$ s.e.m. of technical replicates; *P* values were determined using a two-tailed unpaired Student's *t*-test. Data represent three (**a**, **b**, **e**-**h**) independent experiments or are pooled from at least three (**c**) independent staining experiments. Source data for **a**, **c**, **e**-**h** are provided as a Source Data file.

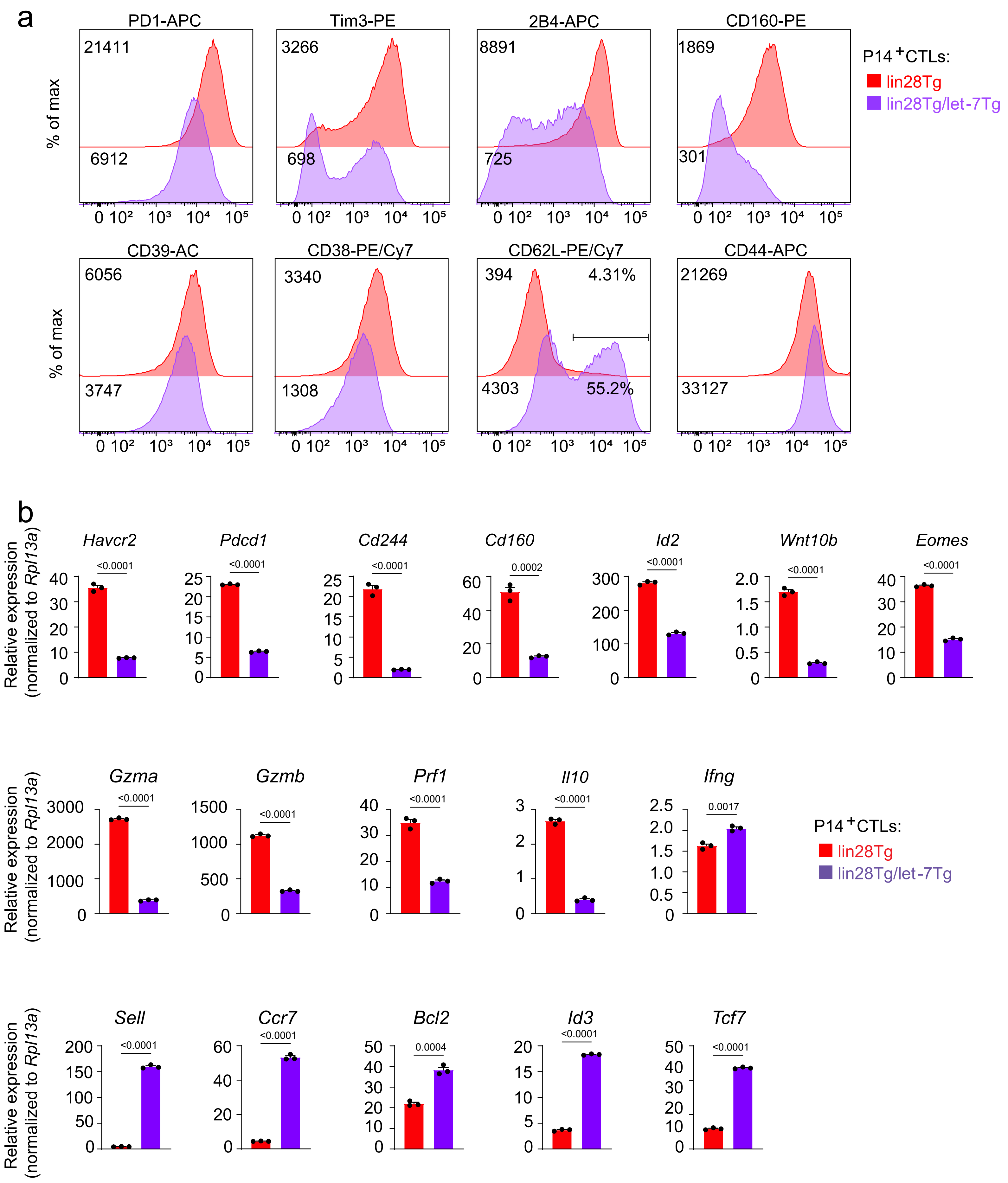

Supplementary Fig. 4

Supplementary Fig. 4.

a, Representative FACS analysis of the surface expression of terminal differentiation and memory markers in P14^{+}lin28Tg and P14^{+}lin28Tglet-7Tg CTLs. The numbers indicate MFIs and frequency (for CD62L staining). **b**, Quantitative RT-PCR analysis of the expression of genes involved in terminal differentiation and memory formation P14+ lin28Tg and lin28Tglet-7Tg CTLs. Data represent two independent experiments, data in **b** are the mean $\pm$ s.e.m. of technical triplicates; *P* values were determined using a two-tailed unpaired Student's *t*-test. Source data for **b** are provided as a Source Data file.

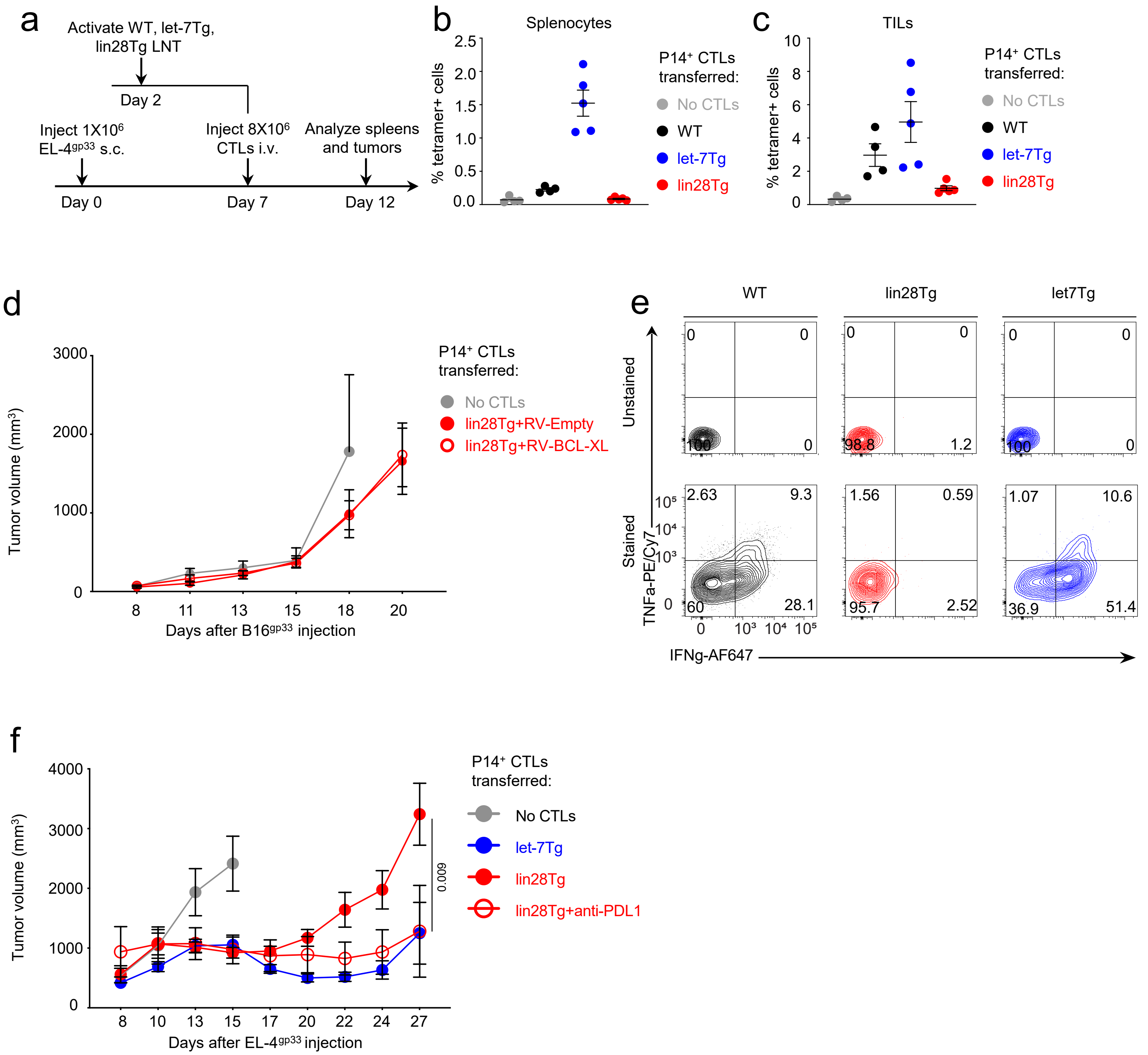

Supplementary Fig. 5

Supplementary Fig. 5.

a-**c**, Experimental design (**a**), quantification of the frequency of donor P14+ CTLs recovered from spleen (**b**) and TILs (**c**) of WT mice s.c. injected with EL-4^{gp33} tumor cells and adoptively transferred with WT (n=4 mice), let-7Tg (n=5 mice) or lin28Tg (n=5 mice) P14^{+} CTLs. Grey color represents control group that received no CTLs (n=4 mice). **d**, Tumor growth curves in WT CD45.1 mice s.c. injected with B16^{gp33} tumor cells and adoptively transferred with lin28Tg P14^{+} CTLs infected with either empty (n=7 mice) or Bcl2l1-expressing (n=12 mice) RVs. Grey color represents control group that received no CTLs (n=5). **e**, Representative FACS analysis of TNFα and IFNγ in TILs of WT CD45.1 mice s.c. injected with B16^{gp33} tumor cells and adoptively transferred with donor WT, let-7Tg or lin28Tg P14^{+} CTLs. **f**, Tumor growth curves in WT mice (n=5 mice per group) s.c. injected with EL4^{gp33} tumor cells and adoptively transferred with lin28Tg P14^{+} CTLs with or without anti-PDL1 treatment and let-7Tg P14^{+} CTLs. Grey color represents control group that received no CTLs. *P* value for tumor growth curves was determined using two-way ANOVA with Sidak's multiple comparison test. Data represent two (**a**-**d**, **f**) independent experiments or three (**e**) independent staining experiments. Source data for **b**-**d**, **f** are provided as a Source Data file.

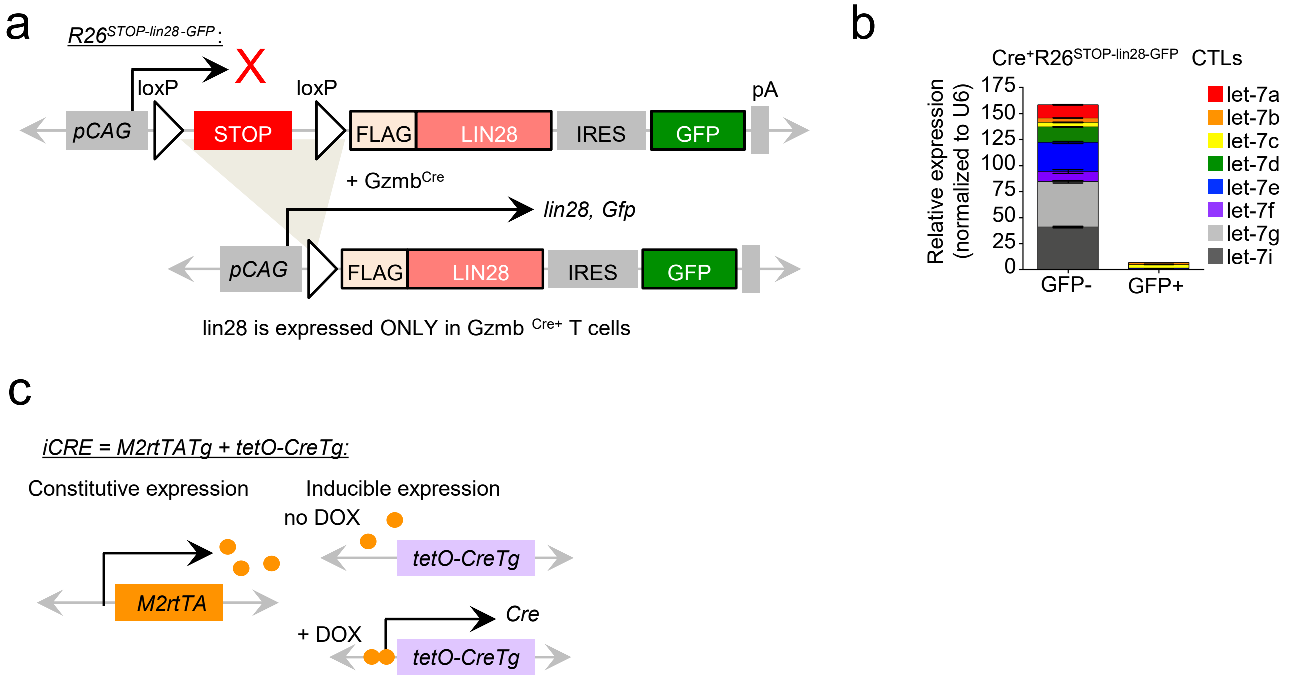

Supplementary Fig. 6

Supplementary Fig. 6.

a, Schematic of GzmbCre^{+}R26$^{STOP\text{-}Lin28\text{-}GFP}$ mice. Cre deletes STOP cassette to induce the expression of Lin28 and the GFP reporter. **b**, Quantitative RT-PCR analysis showing no let-7 expression in CTLs from GzmbCre^{+}R26$^{STOP\text{-}Lin28\text{-}GFP}$ mice. Data represent one experiment and are the mean $\pm$ s.e.m. of technical triplicates. **c**, Doxycycline-inducible Cre (iCre = tetO-CreTg M2rtTATg) is only expressed when dox is present, such that when crossed with R26$^{STOP\text{-}Lin28\text{-}GFP}$ mice, cells will only express Lin28 and GFP in the presence of doxycycline. Source data for **b** are provided as a Source Data file.

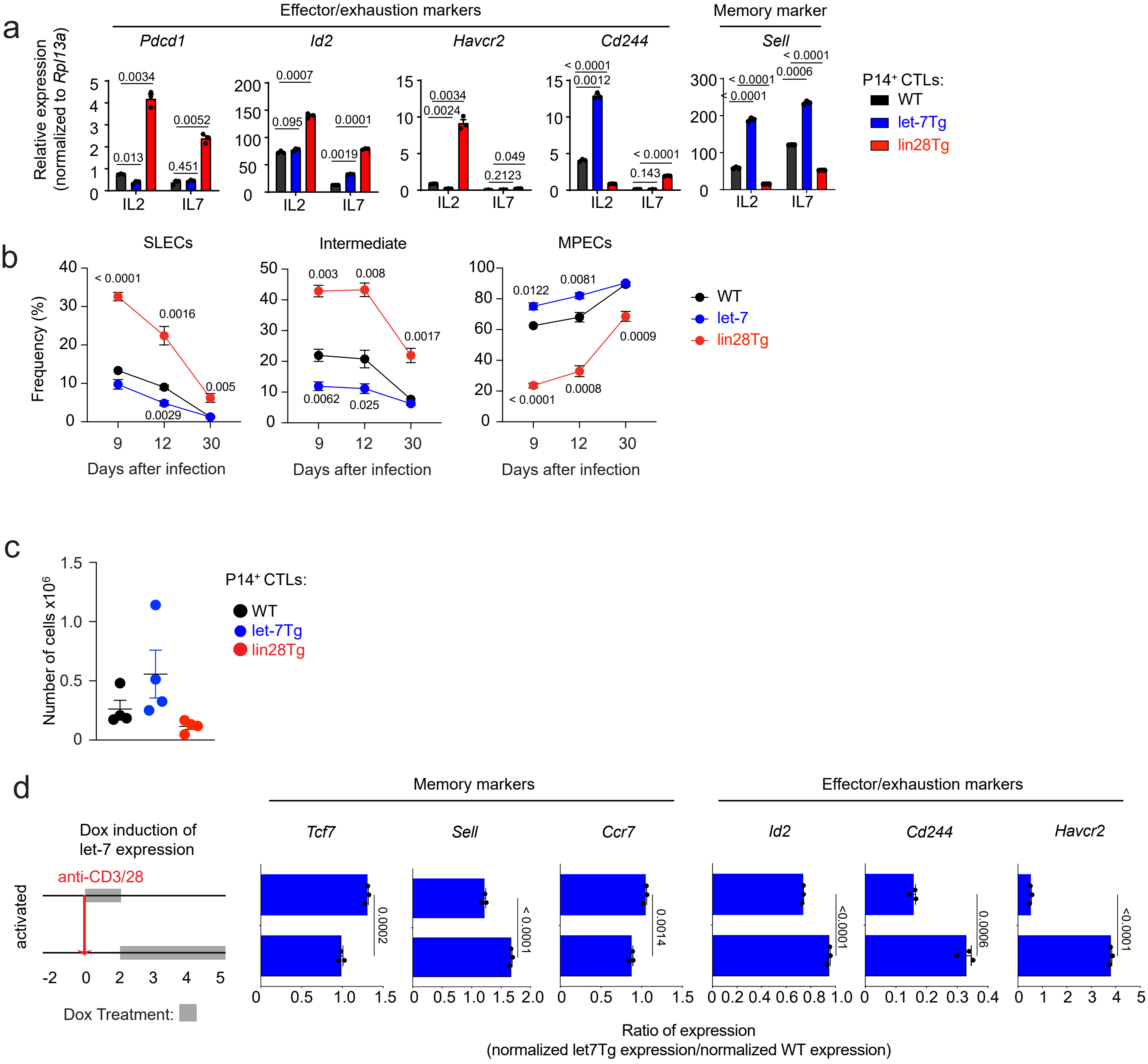

Supplementary Fig. 7

Supplementary Fig. 7.

a, Quantitative RT-PCR analysis of the expression of genes involved in terminal differentiation or memory formation in indicated P14$^+$ CTLs after culture with IL-2 or IL2+IL-7. **b**, Frequencies of KLRG1$^+$CD127$^-$ (SLECs), KLRG1$^+$CD127$^+$ (intermediate) and KLRG1$^-$CD127$^+$ (MPECs) populations plotted over time after *Lm-gp33* infection from the experiment in Figure 3c **c**, Number of P14+ WT, lin28Tg and let-7Tg CTLs recovered from spleens of mice on day 30 after *Lm-gp33* infection from the experiment in Figure 3c. **d**, Quantitative RT-PCR analysis of the expression of genes involved in terminal differentiation or memory formation in let-7Tg P14$^+$ CTLs with addition of dox at indicated time points (grey bars) presented as the ratio between normalized expression in let-7Tg P14$^+$ CTLs and WT P14$^+$ CTLs from Figure 3d. Data are the means $\pm$ s.e.m. of technical triplicates; *P* values were determined using a two-tailed unpaired Student's *t*-test. Data represent two (**a-d**) independent experiments. Source data for **a-d** are provided as a Source Data file.

a

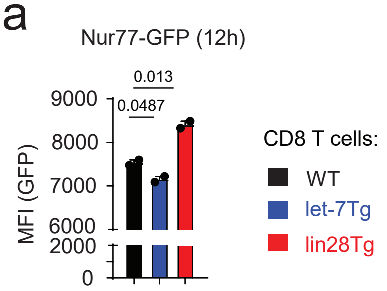

b

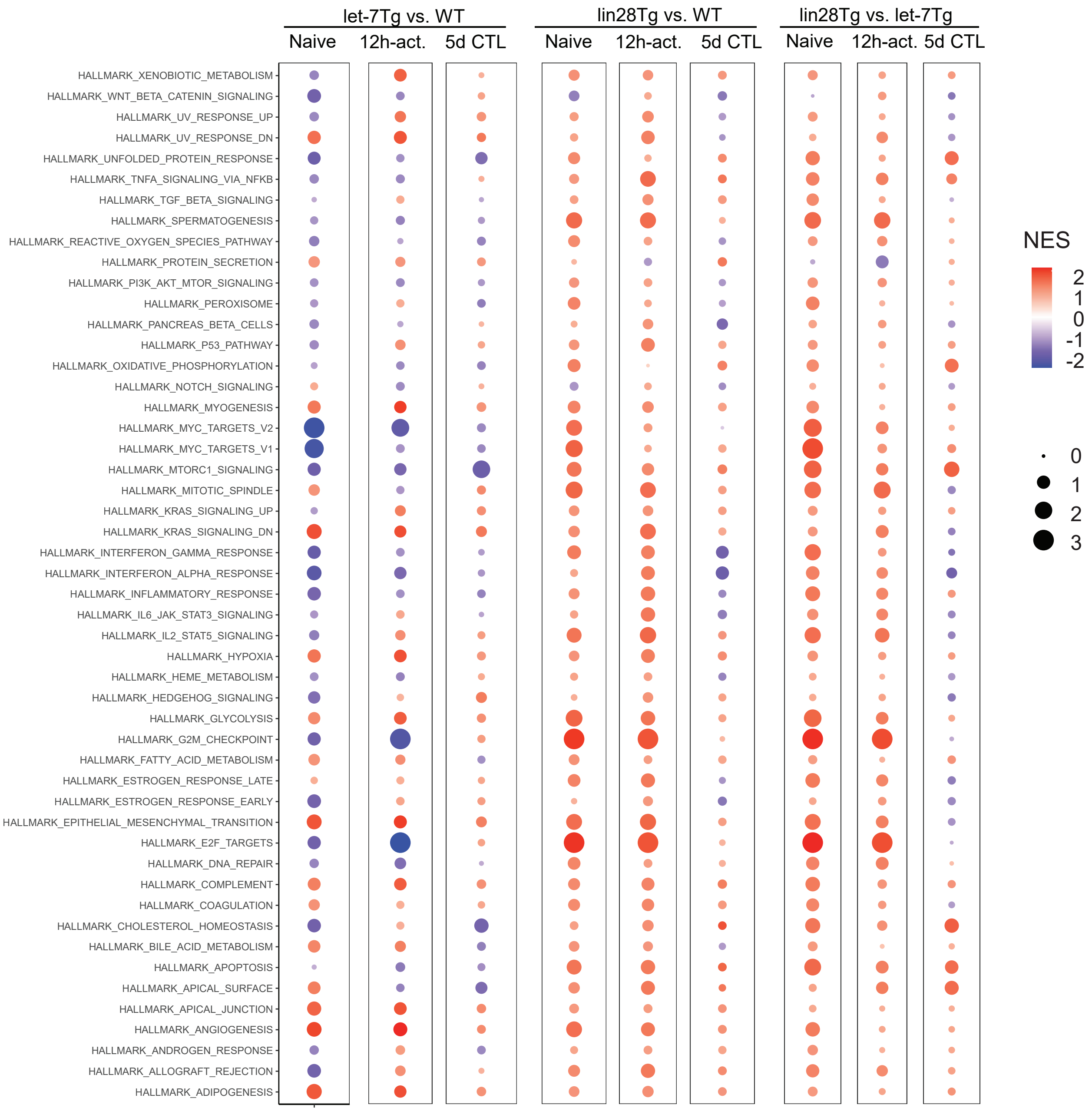

Supplementary Fig. 8

Supplementary Fig. 8.

a, MFIs of Nur77-GFP reporter in P14$^+$ CD8 T cells from Nur77^{GFP}, let-7TgNur77^{GFP} and lin28TgNur77^{GFP} mice activated for 12h with anti-CD3/anti-CD28. Data are the means $\pm$ s.e.m. of biological replicates and are from one experiment; *P* values were determined using a two-tailed unpaired Student's *t*-test. **b**, Full gene set enrichment analysis of 50 Hallmark pathways upregulated and downregulated in P14$^+$ naive, 12h-activated and 5-day differentiated CD8 T cells from WT, lin28Tg and let-7Tg mice. RNAseq data are from Figure 1 and Figure 4. Source data for **a** are provided as a Source Data file.

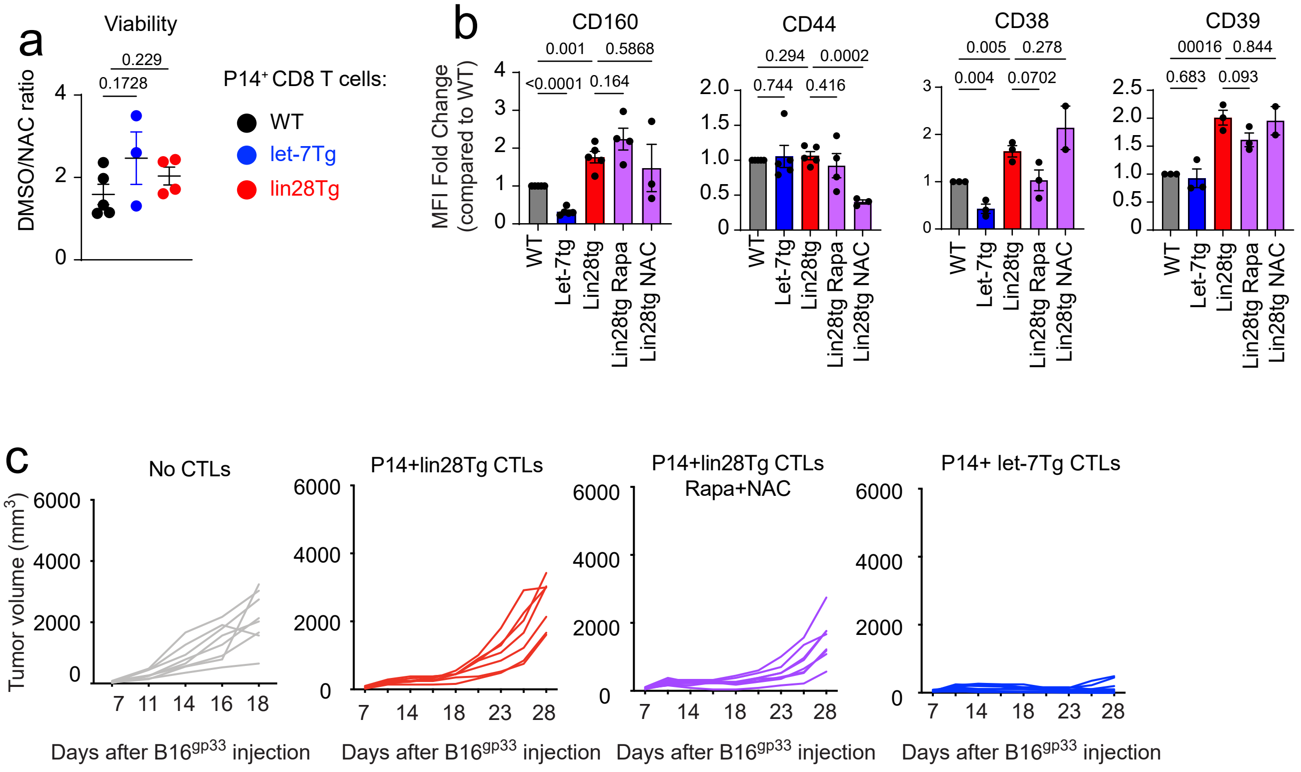

Supplementary Fig. 9

Supplementary Fig. 9.

a, Viability of CTLs on day 5 after NAC treatment presented as the ratio between the cell numbers in DMSO cultures and the cell numbers in NAC-containing cultures. **b**, Quantification of MFIs for CD160, CD44, CD38 and CD39 in indicated cells and presented as a fold change relative to WT. **c**, Individual tumor growth curves in WT mice s.c. injected with $B16^{gp33}$ tumor cells and adoptively transferred with $P14^{+}$ CTLs from lin28Tg mice differentiated with (n=8) or without (n=7) rapamycin+NAC or let-7Tg mice (n=10). Grey color represents control group that received no CTLs (n=8). Data in **a,b** are the means $\pm$ s.e.m. of independent biological replicates. In **a** n=5 for WT, n=4 for lin28Tg and n=3 for let-7Tg CTLs. In **b** for CD160 and CD44 staining n=5 for WT, let-7Tg and lin28Tg and n=3 for lin28TgRapa and lin28TgNAC; for CD38 and CD39 staining n=3 for WT, let-7Tg, lin28Tg and lin28TgRapa and n=2 for lin28TgNAC. Source data for **a-c** are provided as a Source Data file.

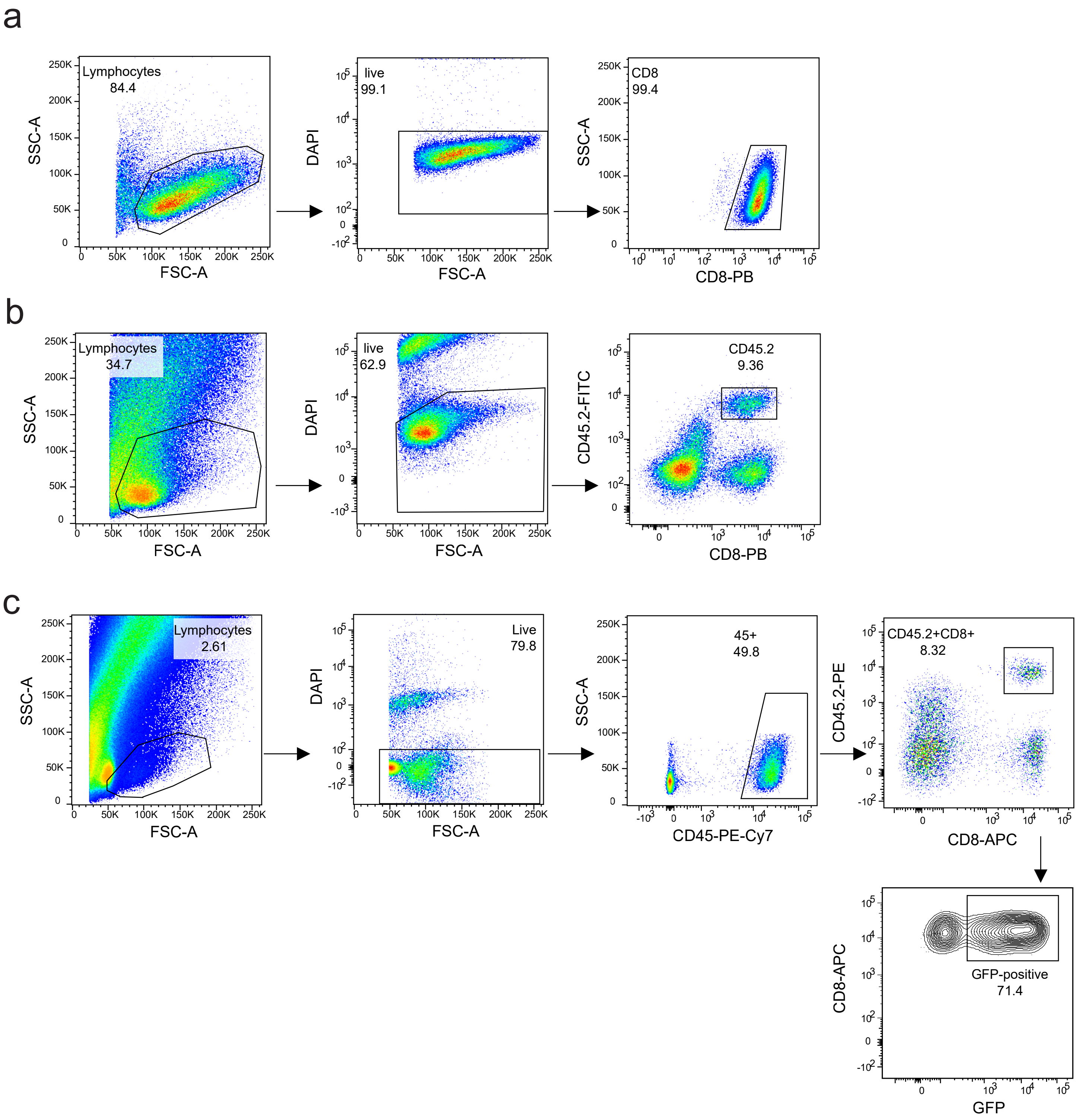

Supplementary Fig. 10

Supplementary Fig. 10.

a, Gating strategy for FACS analysis of live CD8+ T cells from in vitro cultures or blood/spleen (related to Fig. 1i-j; 3c; 5b-e; 5g-h; S3b, c, g, h; S4a; S7b, c, S9a, b). **b**, Gating strategy for FACS analysis of live donor CD45.2+CD8+ T cells from TILs or spleens of tumor bearing mice (related to Fig. 2a-b, d; S5c, e). **c**, Gating strategy for FACS analysis of live donor CD45.2+GFP+CD8+ T cells from TILs or spleens of tumor bearing mice (related to Fig. 2c). For all experiments, cells were identified by single cell gating on FSC-A/FSC-H before acquisition and only singlets were acquired.
