# Supplementary material for: Let-7 enhances murine anti-tumor CD8 T cell responses by promoting memory and antagonizing terminal differentiation
Source: Nat Commun. 2023 Sep 11;14:5585. doi: 10.1038/s41467-023-40959-7 (PMC10495470; doi:10.1038/s41467-023-40959-7)
Supplement: Supplementary file 3 — Description of Additional Supplementary Files [file 41467_2023_40959_MOESM3_ESM.pdf]

Description of Additional Supplementary Files

Supplementary Data 1:
List of top 177 upregulated and downregulated differentially expressed genes in day 5 $P14^+$ CTLs from either WT, lin28Tg or let-7Tg mice.

Supplementary Data 2:
List of cluster-4 genes (from Figure 1e) upregulated in naive $P14^+$ CD8 T cells from lin28Tg or let-7Tg mice.

Supplementary Data 3:
List of cluster-4 genes (from Figure 1e) upregulated in 12h-activated $P14^+$ cells from lin28Tg or let-7Tg mice.

Supplementary Data 4:
List of let-7 target genes in $P14^+$ naïve, 12h-activated and 5-day differentiated CD8 T cells from cluster-1 (from Figure 1e).

Supplementary Data 5:
List of SYBR Green primers and Taqman assays
