# Supplementary material for: Let-7 enhances murine anti-tumor CD8 T cell responses by promoting memory and antagonizing terminal differentiation
Source: Nat Commun. 2023 Sep 11;14:5585. doi: 10.1038/s41467-023-40959-7 (PMC10495470; doi:10.1038/s41467-023-40959-7)
Supplement: Supplementary file 9 — Reporting Summary [file 41467_2023_40959_MOESM9_ESM.pdf]

nature portfolio

Corresponding author(s): Elena Pobezinskaya, Leonid Pobezinsky

Last updated by author(s): Aug 2, 2023

Reporting Summary

Nature Portfolio wishes to improve the reproducibility of the work that we publish. This form provides structure for consistency and transparency in reporting. For further information on Nature Portfolio policies, see our Editorial Policies and the Editorial Policy Checklist.

Statistics

For all statistical analyses, confirm that the following items are present in the figure legend, table legend, main text, or Methods section.

n/a	Confirmed	
☐	☒	The exact sample size (*n*) for each experimental group/condition, given as a discrete number and unit of measurement
☐	☒	A statement on whether measurements were taken from distinct samples or whether the same sample was measured repeatedly
☐	☒	The statistical test(s) used AND whether they are one- or two-sided<br>*Only common tests should be described solely by name; describe more complex techniques in the Methods section.*
☒	☐	A description of all covariates tested
☐	☒	A description of any assumptions or corrections, such as tests of normality and adjustment for multiple comparisons
☐	☒	A full description of the statistical parameters including central tendency (e.g. means) or other basic estimates (e.g. regression coefficient) AND variation (e.g. standard deviation) or associated estimates of uncertainty (e.g. confidence intervals)
☒	☐	For null hypothesis testing, the test statistic (e.g. *F*, *t*, *r*) with confidence intervals, effect sizes, degrees of freedom and *P* value noted<br>*Give P values as exact values whenever suitable.*
☒	☐	For Bayesian analysis, information on the choice of priors and Markov chain Monte Carlo settings
☒	☐	For hierarchical and complex designs, identification of the appropriate level for tests and full reporting of outcomes
☒	☐	Estimates of effect sizes (e.g. Cohen's *d*, Pearson's *r*), indicating how they were calculated

Our web collection on statistics for biologists contains articles on many of the points above.

Software and code

Policy information about availability of computer code

Data collection	LSRFortessa (Becton Dickinson), QuantStudio 6 Flex Real-time system (Applied Biosciences), Seahorse XFe96 Analyzer (Agilent technologies)
Data analysis	FlowJo 10.8.1, Prism 9 (Graphpad software), Adobe Illustrator (version 27.7), RStudio (R version 4.2.1), GSEA (v4.2.3), Bowtie (v2.0.6), TopHat (v2.0.9), HTSeq (v0.6.1). R packages DESeq2 (v2_1.6.3), PCAtools (v2.8.0), car (v3.1-2), RColorBrewer (v1.1-3), ggplot2 (v3.4.2), EnhancedVolcano (v1.14.0), pheatmap (v1.0.12), dendextend (v1.17.1),Targetscan (version 8.0)

For manuscripts utilizing custom algorithms or software that are central to the research but not yet described in published literature, software must be made available to editors and reviewers. We strongly encourage code deposition in a community repository (e.g. GitHub). See the Nature Portfolio guidelines for submitting code & software for further information.

Data

Policy information about availability of data

All manuscripts must include a data availability statement. This statement should provide the following information, where applicable:

- Accession codes, unique identifiers, or web links for publicly available datasets
- A description of any restrictions on data availability
- For clinical datasets or third party data, please ensure that the statement adheres to our policy

The sequencing data that support the findings of this study have been deposited in the National Center of Biotechnology Information Gene Expression Omnibus (GEO) and are accessible through the accession number GSE232541 (https://www.ncbi.nlm.nih.gov/geo/query/acc.cgi?acc=GSE232541). The gene sets used for GSEA in this study are accessible from MSigDB under GEO accession codes GSE8678 (GSE8678_IL7R_LOW_VS_HIGH_EFF_CD8_TCELL_UP (gsea-msigdb.org)) and

GSE10239 (GSE10239_MEMORY_VS_DAY4.5_EFF_CD8_TCELL_UP (gsea-msigdb.org)). The list of target genes by miRNA were obtained from the TargetScan database (TargetScanMouse 8.0). Source data are provided as a Source Data file.

Research involving human participants, their data, or biological material

Policy information about studies with human participants or human data. See also policy information about sex, gender (identity/presentation), and sexual orientation and race, ethnicity and racism.

Reporting on sex and gender	N/A
Reporting on race, ethnicity, or other socially relevant groupings	N/A
Population characteristics	N/A
Recruitment	N/A
Ethics oversight	N/A

Note that full information on the approval of the study protocol must also be provided in the manuscript.

Field-specific reporting

Please select the one below that is the best fit for your research. If you are not sure, read the appropriate sections before making your selection.

- [x] Life sciences
- [ ] Behavioural & social sciences
- [ ] Ecological, evolutionary & environmental sciences

For a reference copy of the document with all sections, see nature.com/documents/nr-reporting-summary-flat.pdf

Life sciences study design

All studies must disclose on these points even when the disclosure is negative.

Sample size	The sample size for each experiment is indicated in the figure legends. No statistical method was used to predetermine the sample size for experiments. The minimum sample size was chosen to reach statistical significance compared to control.
Data exclusions	No data was excluded from analyses.
Replication	For all experiments, at least three replicates were analyzed in at least two independent experiments. Experimental findings were reliably reproduced.
Randomization	Mice were allocated to groups based on genotype. For in vivo tumor experiments host mice were age and sex-matched and were randomly assigned to each group.
Blinding	No blinding was used as scoring methods were not subjecvtive but based on quantitative analyses

Reporting for specific materials, systems and methods

We require information from authors about some types of materials, experimental systems and methods used in many studies. Here, indicate whether each material, system or method listed is relevant to your study. If you are not sure if a list item applies to your research, read the appropriate section before selecting a response.

Materials & experimental systems

n/a	Involved in the study	
[ ]	[x]	Antibodies
[ ]	[x]	Eukaryotic cell lines
[x]	[ ]	Palaeontology and archaeology
[ ]	[x]	Animals and other organisms
[x]	[ ]	Clinical data
[x]	[ ]	Dual use research of concern
[x]	[ ]	Plants

Methods

n/a	Involved in the study	
[x]	[ ]	ChIP-seq
[ ]	[x]	Flow cytometry
[x]	[ ]	MRI-based neuroimaging

Antibodies

Antibodies used	CD8α-Pacific Blue (53-6.7), PRID: AB_493425 (BioLegend, Cat#100725)

CD8α-APC/Cy7 (53-6.7), PRID: AB_312753 (BioLegend, Cat#100714)
CD8α-APC (53-6.7), PRID: AB_312751 (BioLegend, Cat#100712)
CD4-Pacific Blue (RM4-5), PRID: AB_493374 (BioLegend, Cat# 100531)
CD44-FITC (IM7), PRID: AB_493684 (BioLegend, Cat#103021)
CD45-PE/Cy7 (30-F11), PRID: AB_312979 (BioLegend, Cat#103114)
CD45.2-FITC (104), PRID: AB_313443 (BioLegend, Cat#109806)
CD45.2-PE (104), PRID: AB_313445 (BioLegend, Cat#109808)
CD62L-PE/Cy7 (MEL-14), PRID: AB_313103 (BioLegend, Cat#104418)
KLRG1-APC (2F1/KLRG1), PRID: AB_10641560 (BioLegend, Cat#138412)
CD127-bio (A7R34), PRID: AB_2126118 (BioLegen,d Cat#135006)
PD1-bio (29F.1A12), PRID: AB_10640124 (BioLegend, Cat# 135212)
Tim-3-PE (RMT3-23), PRID: AB_345378 (BioLegend, Cat# 119704)
2B4-bio (m2B4(B6)458.1), PRID: AB_1626222 (BioLegend, Cat# 133506)
CD38-PE/Cy7 (90), PRID: AB_2275531 (BioLegend, Cat# 102718)
CD39-APC (Duha59), PRID: AB_2750320 (BioLegend, Cat# 143810)
CD27-APC (LG.3A10), PRID: AB_1236460 (BioLegend, Cat# 124211)
CXCR3-BV650 (CXCR3-173), PRID: AB_2563160 (BioLegend, Cat#126531)
Phospho-Erk1/2-PE (6B8B69), PRID: AB_2629704 (BioLegend, Cat# 369505)
TNFa-PE/Cy7 (MP6-XT22), PRID: AB_2256076 (BioLegend, Cat# 506324)
IL-10-PE (JES5-16E3), PRID: AB_315362 (BioLegend, Cat# 505008)
CD28-PE (37.51), RRID: AB_394766 (BD Biosciences, Cat# 553297)
CD160-PE (CNX46-3), RRID: AB_1210733 (Thermo Fisher Scientific, Cat# 12-1601-81)
IFNg-APC (XMG 1.2), RRID: AB_469503 (Thermo Fisher Scientific, Cat# 17-7311-81)
TCF-1-AF647 (S33-966), RRID: AB_2869823 (BD Biosciences, Cat# 566693)
Foxo1-PE (C29H4), PRID: AB_2798437 (Cell Signaling, Cat# 14262)
phospho-Akt-PE (D25E6), PRID: AB_2798327(Cell Signaling, Cat# 13842)
phospho-Akt-APC (D9E), PRID: AB_2797780 (Cell Signaling, Cat# 11962)
Phospho-S6-PE (D57.2.2E), PRID: AB_10694989 (Cell Signaling, Cat# 5316)

anti-PD-L1 (10F.9G2) , PRID: AB_10949073 (BioXCell, Cat# BE0101)
Rat IgG2b, κ isotype control (LTF-2), PRID: AB_1107780 (BioXCell, Cat# BE0090)

Validation

All antibodies are widely used by research community, are commercially available and have been validated using flow cytometry by the manufacturers, in previous reports and by ourselves in our experiments. Validation and relevant citations can be found on the manufacturer's websites (BioLegend, Thermo Fisher Scientific, BD Biosciences and Cell Signaling):

https://www.biolegend.com/en-us/products/pacific-blue-anti-mouse-cd8a-antibody-2856
https://www.biolegend.com/en-us/products/apc-cyanine7-anti-mouse-cd8a-antibody-2269
https://www.biolegend.com/en-us/products/apc-anti-mouse-cd8a-antibody-150
https://www.biolegend.com/en-us/products/pacific-blue-anti-mouse-cd4-antibody-2855
https://www.biolegend.com/en-us/products/fitc-anti-mouse-human-cd44-antibody-314
https://www.biolegend.com/en-us/products/pe-cyanine7-anti-mouse-cd45-antibody-1903
https://www.biolegend.com/en-us/products/fitc-anti-mouse-cd45-2-antibody-6
https://www.biolegend.com/en-us/products/pe-anti-mouse-cd45-2-antibody-7
https://www.biolegend.com/en-us/products/pe-cyanine7-anti-mouse-cd62l-antibody-1922
https://www.biolegend.com/en-us/products/apc-anti-mouse-human-klrg1-mafa-antibody-6866
https://www.biolegend.com/en-us/products/biotin-anti-mouse-cd127-il-7ralpha-antibody-6269
https://www.biolegend.com/en-us/products/biotin-anti-mouse-cd279-pd-1-antibody-6723
https://www.biolegend.com/en-us/products/pe-anti-mouse-cd366-tim-3-antibody-2657
https://www.biolegend.com/en-us/products/biotin-anti-mouse-cd244-2-2b4-b6-alloantigen-antibody-5918
https://www.biolegend.com/en-us/products/pe-cyanine7-anti-mouse-cd38-antibody-3926
https://www.biolegend.com/en-us/products/apc-anti-mouse-cd39-antibody-16388
https://www.biolegend.com/en-us/products/apc-anti-mouse-rat-human-cd27-antibody-4395
https://www.biolegend.com/en-us/products/brilliant-violet-650-anti-mouse-cd183-cxcr3-antibody-9384
https://www.biolegend.com/en-us/products/pe-anti-erk1-2-phospho-thr202-tyr204-antibody-13590
https://www.biolegend.com/en-us/products/pe-cyanine7-anti-mouse-tnf-alpha-antibody-5866
https://www.biolegend.com/en-us/products/pe-anti-mouse-il-10-antibody-944

https://www.thermofisher.com/antibody/product/CD160-Antibody-clone-eBioCNX46-3-CNX46-3-Monoclonal/12-1601-81
https://www.thermofisher.com/antibody/product/IFN-gamma-Antibody-clone-XMG1-2-Monoclonal/17-7311-8

https://www.bdbiosciences.com/en-us/products/reagents/flow-cytometry-reagents/research-reagents/single-color-antibodies-ruo/alexa-fluor-647-mouse-anti-tcf-7-tcf-1.566693
https://www.bdbiosciences.com/en-us/products/reagents/flow-cytometry-reagents/research-reagents/single-color-antibodies-ruo/pe-hamster-anti-mouse-cd28.553297

https://www.cellsignal.com/products/antibody-conjugates/foxo1-c29h4-rabbit-mab-pe-conjugate/14262
https://www.cellsignal.com/products/antibody-conjugates/phospho-akt-thr308-d25e6-xp-rabbit-mab-pe-conjugate/13842
https://www.cellsignal.com/products/antibody-conjugates/phospho-akt-ser473-d9e-xp-rabbit-mab-apc-conjugate/11962

https://www.cellsignal.com/products/antibody-conjugates/phospho-s6-ribosomal-protein-ser235-236-d57-2-2e-xp-rabbit-mab-pe-conjugate/5316

https://bioxcell.com/invivomab-anti-mouse-pd-l1-b7-h1-be0101?gad=1&gclid=CjwKCAjwt52mBhB5EiwA05YKo4VE_Q0uig7_8WMmLe3HxmKTHZ-O_Jo38OFY8XAfJV0-9lwT4DRSlxoCTYQQAvD_BwE
https://bioxcell.com/invivomab-rat-igg2b-isotype-control-anti-keyhole-limpet-hemocyanin-be0090

Eukaryotic cell lines

Policy information about cell lines and Sex and Gender in Research

Cell line source(s)	B16-F10 (ATCC CRL-6475), MC57G (ATCC CRL-2295), EL4 (ATCC TIB-39).
Authentication	Functional authentication: cells were injected subcutaneously into mice and tumor progression was followed
Mycoplasma contamination	Cells were tested for mycoplasma by the manufacturer
Commonly misidentified lines (See ICLAC register)	No commonly misidentified cell lines were used in this study

Animals and other research organisms

Policy information about studies involving animals; ARRIVE guidelines recommended for reporting animal research, and Sex and Gender in Research

Laboratory animals	C57BL/6J (stock no. 000664), Jackson Laboratory<br>B6.SJL- PtprcaPepcb/ BoyJ (stock no. 002014), Jackson Laboratory<br>B6.Cg-Rag2tm1.1Cgn/J (stock no. 008449), Jackson Laboratory<br>Nur77GFP: C57BL/6-Tg(Nr4a1-EGFP/cre)820Khog/J (stock no. 016617), Jackson Laboratory<br>iCre: Tg(tetO-cre)1Jaw/J (stock no. 006224), Jackson Laboratory<br>GzmbCre+ (B6;FVB-Tg(GZMB-cre)1Jcb/J) mice were a generous gift from Dr. Rodriguez<br>P14+Lin28Tg and P14+let-7Tg mice on a Rag2-/- background, Wells et al, 2017<br>R26STOP-Lin28-GFP, generated for this paper<br>GzmbCre+R26STOP-Lin28-GFP, generated for this paper<br>iCre R26STOP-Lin28-GFP, generated for this paper<br>WTNur77GFP, let-7TgNur77GFP and lin28TgNur77GFP, generated for this paper
Wild animals	No wild animals were used in this study.
Reporting on sex	Male mice (7-8 weeks old) were used for all in vivo tumor experiments except for one experiment in Fig.5i,j where 8-week-old female mice were used. Both male and female mice (6-10-week-old) were used in in vitro CTL experiments without any bias.
Field-collected samples	No field-collected samples were used in this study.
Ethics oversight	This study was performed in accordance with the recommendations in the Guide for the Care and Use of Laboratory Animals of the National Institutes of Health. All animals were handled according to approved institutional animal care and use committee (IACUC) protocols (#2186, 2955) of the University of Massachusetts.

Note that full information on the approval of the study protocol must also be provided in the manuscript.

Flow Cytometry

Plots

Confirm that:

- [x] The axis labels state the marker and fluorochrome used (e.g. CD4-FITC).
- [x] The axis scales are clearly visible. Include numbers along axes only for bottom left plot of group (a 'group' is an analysis of identical markers).
- [x] All plots are contour plots with outliers or pseudocolor plots.
- [x] A numerical value for number of cells or percentage (with statistics) is provided.

Methodology

Sample preparation	Single cell suspensions were prepared in cold FACS buffer (PBS+0.5% BSA+0.01% sodium azide)
Instrument	LSRFortessa (Becton Dickinson)

Software	FlowJo 10.8.1
Cell population abundance	>95% live cells were analyzed in all experiments
Gating strategy	For all experiments, cells were identified by single cell gating on FSC-A/FSC-H. Then dead cells were removed based on DAPI or Live/Dead fixable Aqua Dead Cell Stain Kit staining.

☒ Tick this box to confirm that a figure exemplifying the gating strategy is provided in the Supplementary Information.
